# Sex-Specific Association of Left Ventricular Function With Mortality in Severe Mitral Regurgitation

**DOI:** 10.1001/jamanetworkopen.2025.2420

**Published:** 2025-03-31

**Authors:** Soongu Kwak, Byung Joo Sun, Sahmin Lee, Jun-Bean Park, Hyung-Kwan Kim, Yong-Jin Kim, Jong-Min Song, Seung-Pyo Lee, Dae-Hee Kim

**Affiliations:** 1 Division of Cardiology, Department of Internal Medicine, Seoul National University Hospital, Seoul, South Korea; 2 Division of Cardiology, Department of Internal Medicine, Asan Medical Center, University of Ulsan College of Medicine, Seoul, South Korea; 3 Department of Internal Medicine, Seoul National University College of Medicine, Seoul, South Korea; 4 Center for Precision Medicine, Seoul National University Hospital, Seoul, South Korea

## Abstract

**Importance:**

The association between left ventricular (LV) systolic dysfunction and adverse outcomes in patients with severe degenerative mitral regurgitation (MR) may vary by sex.

**Objective:**

To investigate the sex-specific association of LV systolic function with mortality in patients with severe degenerative MR.

**Design, Setting, and Participants:**

This retrospective cohort study included consecutive patients who underwent surgery for severe degenerative MR at 2 tertiary hospitals in South Korea from 2006 to 2020. Preoperative LV ejection fraction (LVEF) and LV global longitudinal strain (LV-GLS) were measured, with absolute LV-GLS values reported. Patients were stratified by LVEF (≤55%, >55% to ≤60%, and >60%) and LV-GLS (<19.9%, ≥19.9% to <23.4%, and ≥23.4%) tertiles. Mortality status was verified through December 2023, with a median (IQR) follow-up duration of 8.2 (5.3-12.2) years. Data analysis was conducted in March 2024.

**Exposures:**

Surgical MV repair or replacement.

**Main Outcomes and Measures:**

The primary outcome was all-cause mortality after MV surgery. Restricted cubic spline (RCS) curves visualized the association between LVEF, LV-GLS, and mortality for each sex.

**Results:**

Among 1686 patients, 1088 (64.5%) were men and 598 (35.5%) were women. Women were older than men (median [IQR] age, 62 [51-70] vs 54 [45-63] years) and had higher LVEF and LV-GLS than men did. During the follow-up, 220 (13.0%) deaths occurred (117 men [10.8%]; 103 women [17.2%]). In Kaplan-Meier curves, mortality in men increased in the lowest LVEF (≤55%) and LV-GLS (<19.9%) groups, whereas women exhibited an earlier increase in mortality at higher LVEF (55%-60%) and LV-GLS (19.9%-23.4%) levels. Multivariable Cox analyses showed a higher mortality risk associated with LVEF less than or equal to 55% (adjusted hazard ratio [HR], 3.48; 95% CI, 1.84-6.58; *P* < .001) and 55% to 60% (adjusted HR, 2.21; 95% CI, 1.36-3.58; *P* = .001) compared with LVEF greater than 60% in women, but not in men (*P *for interaction by sex = .02). RCS curves showed an earlier increase in mortality risk at higher LVEF and LV-GLS levels in women. Similar trends were observed in asymptomatic patients.

**Conclusions and Relevance:**

In this cohort study of patients with severe degenerative MR, women had an earlier increase in mortality risk associated with LV systolic dysfunction, suggesting the need to consider sex-specific criteria for early surgery in asymptomatic patients.

## Key Points

**Question:**

Are there sex-specific associations between left ventricular (LV) systolic function and adverse outcomes in patients with severe degenerative mitral regurgitation (MR)?

**Findings:**

In a cohort study of 1686 patients, women had an earlier increase in mortality risk associated with LV systolic dysfunction compared with men. This association was consistent in asymptomatic patients, with women experiencing a more marked mortality risk increase compared with men.

**Meaning:**

These findings suggest that women with severe degenerative MR face higher mortality risks at earlier LV systolic dysfunction stages than men, underscoring the need for sex-specific early intervention criteria.

## Teaser

This cohort study examines sex-specific associations of left ventricular systolic function with mortality among patients with severe degenerative mitral regurgitation.

## Introduction

Severe degenerative mitral regurgitation (MR), primarily caused by mitral valve (MV) prolapse or flail leaflet, is associated with substantial mortality.^1,2,3^ Without intervention, adverse left ventricular (LV) remodeling progresses from volume overload, ultimately leading to impaired systolic function, symptoms, and heart failure.^4^ Therefore, it is crucial to detect the early signs of LV decompensation, especially in asymptomatic patients, and to intervene before such decompensation develops further. LV global longitudinal strain (LV-GLS) is a sensitive imaging marker for myocardial contractile function.^5^ Studies have shown that LV-GLS provides powerful prognostic information in patients with severe MR,^6,7,8^ even in those without symptoms or with preserved LV ejection fraction (LVEF).^9,10^ This imaging biomarker thus has the potential to guide optimal timing of intervention in patients with severe MR.

Sex differences in the prognostic outcomes of LV systolic dysfunction in severe degenerative MR remain unclear. A recent study^11^ demonstrated that women have an increased mortality risk at a lower LV end-systolic dimension compared with men, and mortality associated with decreasing LVEF was higher in women than in men. This finding suggests a potential need for earlier intervention in women. However, available data are limited, and sex-specific information regarding the association between LV-GLS and adverse outcomes is lacking. Such data may help determine the optimal timing of intervention in each sex.

This study aimed to investigate sex-specific associations between LV systolic function, including LVEF and LV-GLS, and long-term mortality in patients with severe degenerative MR. In addition, we focused on asymptomatic patients to provide insights into early intervention based on LV systolic dysfunction in each sex.

## Methods

### Study Population

This retrospective cohort study was conducted at 2 tertiary hospitals in South Korea: Asan Medical Center and Seoul National University Hospital. Consecutive patients who met the following inclusion criteria between 2006 and 2020 were analyzed: (1) severe MR diagnosed on echocardiography and undergoing MV surgery (either repair or replacement), and (2) degenerative MR caused by MV prolapse or flail leaflets. Exclusion criteria included age younger than 18 years, a history of MV surgery or intervention (eg, percutaneous balloon mitral commissurotomy), mitral stenosis of moderate or greater degree, any other severe valvular heart disease, rheumatic or congenital MR, acute MR, and secondary MR. Patients with inadequate echocardiography image quality for measuring LV-GLS were also excluded.

The study complies with the Declaration of Helsinki,^12^ and the local institutional review board at each study center approved the study protocol. The same review boards waived the requirement for written informed consent since anonymized and unidentifiable information was used, in accordance with the exemption criteria for retrospective studies involving deidentified data. This study adheres to the Strengthening the Reporting of Observational Studies in Epidemiology (STROBE) reporting guidelines for cohort studies.

### Data Collection and Variable Definitions

Clinical and echocardiographic data at the time of MV surgery were collected. Symptom status at the time of MV surgery, whether symptomatic or asymptomatic MR confirmed by clinicians, was collected from the medical records. Each symptom, including dyspnea, chest pain, and peripheral edema, was collected. The type of MV surgery was categorized as MV repair or replacement, with the latter including replacements using either a mechanical or a bioprosthetic valve. In addition, information regarding any concurrent coronary artery bypass grafting or surgical atrial ablation procedures was collected.

Transthoracic echocardiography was performed shortly before MV surgery (median [IQR] interval, 22 [6-51] days) following the guidelines.^13,14^ The details of the echocardiography measurements are described in eMethods in Supplement 1. LV ejection fraction was calculated using the biplane Simpson method.

### Left Ventricular Global Longitudinal Strain

LV-GLS was measured for all participants. Echocardiographic images were archived as DICOM (Digital Imaging and Communications in Medicine) files in the central core laboratory located at Asan Medical Center. Experienced technicians, who were blinded to patients’ information and outcomes, conducted the LV-GLS measurements retrospectively using vendor-independent, postprocessing software (TOMTEC-ARENA TTA2.50.00; TomTec Imaging Systems). From 3 apical views (2-chamber, 3-chamber, and 4-chamber views), LV endocardial borders were manually traced at the end of systole, and automatic tracking of the endocardial border was performed throughout the cardiac cycle. The final LV-GLS was calculated as the average of the longitudinal strain values derived from the apical images. LV-GLS measurements were averaged over 3 cardiac cycles for patients with an atrial fibrillation rhythm during the examination. The absolute value of LV-GLS was used in this study for straightforward interpretation.

### Outcome Assessment

Patients were followed up from the date of MV surgery until death or the last recorded follow-up. The primary outcome was all-cause mortality. The latest mortality status was verified in December 2023 by linking all patients to the official national death records provided by Statistics Korea. This method ensured accurate determination of the survival status for all patients without loss to follow-up, even for those who had relocated, thereby enhancing the validity of the outcome.

### Statistical Analysis

Data analysis was conducted in March 2024. The normality assumptions for continuous variables were tested using the Shapiro-Wilk tests. Continuous variables were presented as medians with IQRs when the assumption was not met, and differences between groups were assessed using the Mann-Whitney *U* test. Categorical variables were presented as frequencies with percentages, and differences between groups were evaluated using the χ^2^ test. The proportion of missing values for the main study variables is presented in eFigure 1 in Supplement 1, indicating complete data for LV-GLS and a single missing value for LVEF.

Kaplan-Meier survival curves by LVEF and LV-GLS categories were plotted for each sex, and group comparisons were conducted using the log-rank test. LVEF was divided into 3 categories with 5% intervals (>60%, ≤60% and >55%, and ≤55%), aligning with the guideline-recommended cutoff of 60% for LV decompensation. Because no established cutoff for LV-GLS exists, it was categorized into 3 groups based on tertiles across the entire cohort (≥23.4%, <23.4% and ≥19.9%, and <19.9%). Cox proportional hazards regression analysis was used to evaluate the association between risk factors and mortality, with results presented as hazard ratios (HRs) with 95% CIs. Both continuous and categorical forms of LVEF and LV-GLS were included in the Cox analysis. Multivariable Cox models were adjusted for established risk factors for mortality in severe degenerative MR.^1^ The difference by sex was tested by adding the interaction term to the Cox model.

Restricted cubic spline (RCS) curves with 4 knots were plotted to assess the associations between LVEF and LV-GLS (as continuous variables) and mortality in each sex based on the multivariable Cox models. Estimated HRs with 95% CIs were presented to illustrate these associations. The analyses were repeated for a subgroup of asymptomatic patients, as well as for asymptomatic patients without evidence of obstructive coronary artery disease, defined as those with no history of myocardial infarction or concomitant coronary artery bypass grafting.

Sensitivity analyses were conducted by dividing the patients into 2 time periods (2006-2014 and 2015-2020), with approximately equal patient numbers in each group. In addition, a propensity-matched cohort was analyzed, matched for age, symptoms, and surgical methods, using a caliper width of 0.2 SD of the logit of the propensity score. Covariate balance was assessed using standardized mean differences, with values less than 0.1 considered indicative of negligible imbalance. Kaplan-Meier survival analyses were then repeated to investigate whether similar mortality trends were observed in each sex.

A 2-tailed *P* < .05 was considered statistically significant. Analyses were conducted using R statistical software version 4.3.0 (R Project for Statistical Computing).^15^

## Results

### Baseline Characteristics According to Sex

The study cohort comprised 1686 patients with severe degenerative MR (1088 men [64.5%] and 598 women [35.5%]). Women were significantly older than men (median [IQR] age, 62 [51-70] vs 54 [45-63] years) and had lower body mass index, hemoglobin levels, and estimated glomerular filtration rates (Table 1). The prevalence of atrial fibrillation was higher in men than in women. Regarding the surgical method, most patients underwent MV repair. The frequency of concomitant coronary artery bypass grafting and surgical atrial ablation was similar between the sexes.

**Table 1.** Baseline Characteristics of the Study Patients According to Sex

Characteristics	Patients, No. (%)		*P* value
	Men (n = 1088)	Women (n = 598)	
Age, median (IQR), y	54 (45-63)	62 (51-70)	<.001
Body mass index, median (IQR)^a^	24.6 (22.6-26.8)	23.7 (21.4-26.2)	<.001
Comorbidities			
Hypertension	439 (40.3)	252 (42.1)	.51
Diabetes	102 (9.4)	56 (9.4)	>.99
Atrial fibrillation	400 (36.8)	191 (31.9)	.05
Stroke	24 (2.2)	26 (4.3)	.02
Myocardial infarction	21 (1.9)	10 (1.7)	.85
Symptomatic mitral regurgitation	370 (34.0)	266 (44.5)	<.001
Symptoms			
Dyspnea	307 (28.2)	237 (39.6)	<.001
Chest pain	51 (4.7)	20 (3.3)	.24
Edema	28 (2.6)	23 (3.8)	.19
Palpitation	64 (5.9)	42 (7.0)	.41
Syncope	8 (0.7)	9 (1.5)	.21
Laboratory results, median (IQR)			
Hemoglobin, g/dL	14.3 (13.3-15.0)	12.5 (11.5-13.2)	<.001
Estimated glomerular filtration rate, mL/min/1.73 m^2^	87.2 (74.4-99.0)	86.1 (67.3-98.3)	.01
Mitral regurgitation surgery type			
MV repair	1002 (92.1)	525 (87.8)	<.001
MV replacement (mechanical)	45 (4.1)	20 (3.3)	
MV replacement (bioprosthetic)	41 (3.8)	53 (8.9)	
Concomitant coronary artery bypass grafting	56 (5.1)	31 (5.2)	>.99
Concomitant surgical atrial ablation	357 (32.8)	173 (28.9)	.11
Echocardiography values, median (IQR)			
LV end-systolic diameter, mm	38 (35-42)	35 (32-39)	<.001
LV end-diastolic diameter, mm	61 (57-65)	57 (53-62)	<.001
LV end-systolic volume, mL	61.0 (50.0-76.0)	46.0 (36.0-59.0)	<.001
LV end-systolic volume index, mL/m^2^	33.5 (27.1-41.6)	29.1 (22.6-37.8)	<.001
LV end-diastolic volume, mL	172.0 (142.0-205.0)	133.0 (110.0-162.5)	<.001
LV end-diastolic volume index, mL/m^2^	93.5 (77.3-112.1)	85.7 (69.0-103.6)	<.001
LV ejection fraction, %	63.1 (59.0-67.1)	64.5 (60.3-68.5)	<.001
LV mass index, g/m^2^	135.0 (116.5-155.1)	128.6 (108.8-152.6)	<.001
Left atrial dimension, mm	51.0 (46.0-58.0)	50.0 (45.0-56.0)	<.001
E-wave, m/s	1.12 (0.80-1.40)	1.14 (0.68-1.43)	.70
e’-wave, cm/s	8.0 (6.4-9.6)	7.0 (5.8-9.0)	<.001
E/e’ ratio	15 (11-19)	17 (13-22)	<.001
Tricuspid regurgitation peak velocity, m/s	2.7 (2.4-3.2)	2.9 (2.6-3.4)	<.001
LV global longitudinal strain, %	21.3 (18.5-24.1)	22.1 (19.1-25.0)	.001

Abbreviations: LV, left ventricular; MV, mitral valve.

SI conversion factor: To convert hemoglobin to grams per liter, multiply by 10.

^a^ Body mass index is calculated as weight in kilograms divided by height in meters squared.

The preoperative echocardiographic assessment demonstrated that men had significantly larger LV chamber dimensions and volumes than women (Table 1). LVEF was significantly lower in men than in women (median [IQR], 63.1% [59.0%-67.1%] vs 64.5% [60.3%-68.5%]), as was LV-GLS (median [IQR], 21.3% [18.5%-24.1%] vs 22.1% [19.1%-25.0%]). The distribution of LVEF and LV-GLS according to sex is shown in eFigure 2 in Supplement 1.

### Association of Left Ventricular Systolic Function and Mortality According to Sex

Over a median (IQR) follow-up of 8.2 (5.3-12.2) years, 220 (13.0%) deaths occurred (103 women [17.2%]; 117 men [10.8%]), with significantly lower overall survival in women than in men (eFigure 3 in Supplement 1). When stratified by follow-up duration, mortality rates were similar between sexes during the early period; however, women exhibited higher mortality with longer follow-up compared with men (eTable 1 in Supplement 1). Women had a higher risk of mortality in the univariable Cox analysis (unadjusted HR, 1.58; 95% CI, 1.21-2.06; *P* < .001), but this association lost significance in the multivariable analysis (adjusted HR, 1.08; 95% CI, 0.82-1.43; *P* = .59).

**Figure 1.**
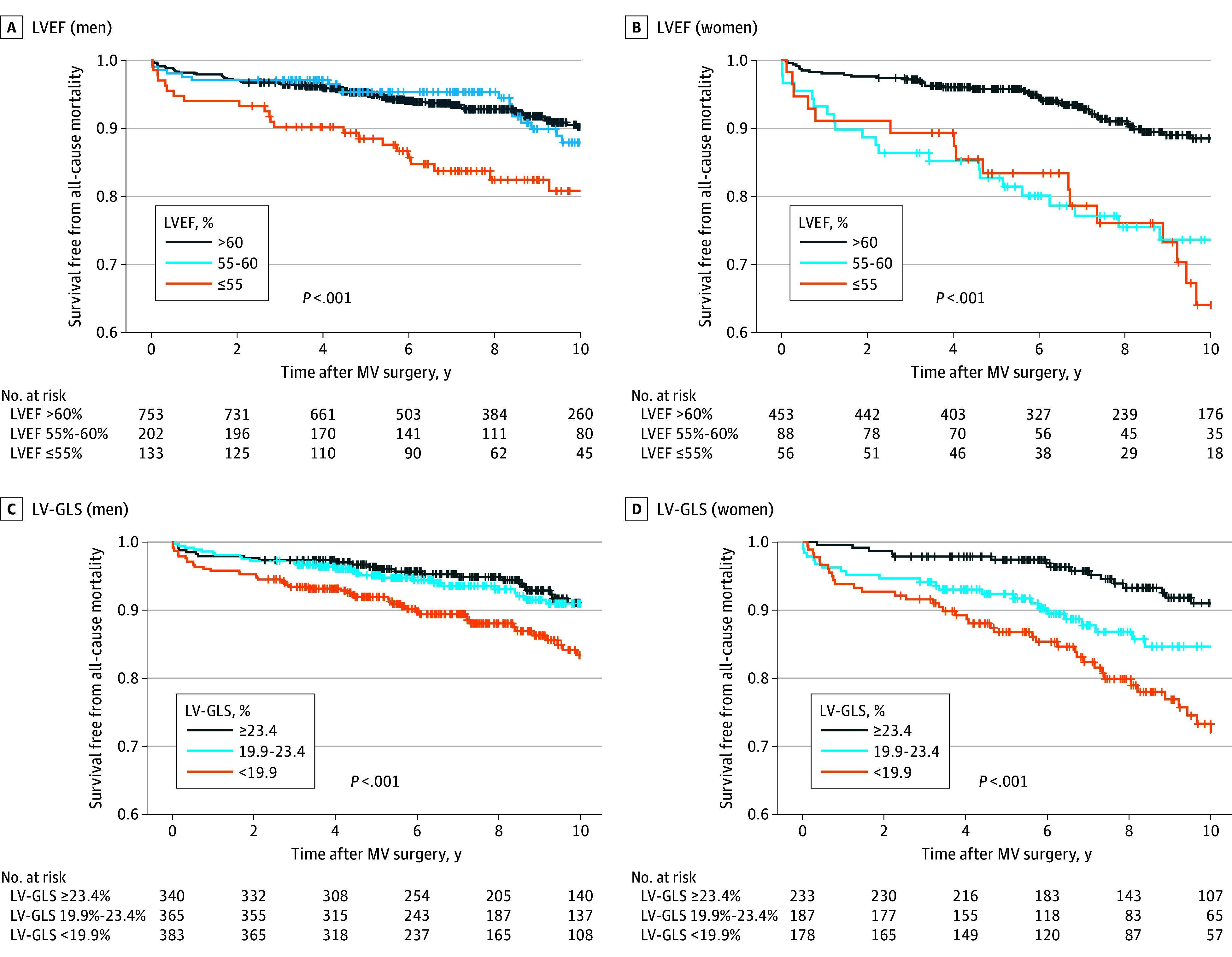
Mortality According to Left Ventricular Systolic Function and Sex A, *P* = .35 for left ventricular ejection fraction (LVEF) 55% to 60%, and *P* < .001 for LVEF less than or equal to 55% (reference, LVEF >60%). B, *P* < .001 for LVEF 55% to 60%, and *P* < .001 for LVEF less than or equal to 55% (reference, LVEF >60%). C, *P* = .80 for left ventricular global longitudinal strain (LV-GLS) 19.9% to 23.4%, and *P* < .001 for LV-GLS less than 19.9% (reference, LV-GLS ≥23.4%). D, *P* = .08 for LV-GLS 19.9% to 23.4%, and *P* < .001 for LV-GLS less than 19.9% (reference, LV-GLS ≥23.4%). MV indicates mitral valve.

When stratified by LVEF, men with an LVEF less than or equal to 55% exhibited the lowest survival rate among the 3 groups, whereas those with an LVEF greater than 60% and 55% to 60% showed similar survival rates (Figure 1A). In contrast, in women, the mortality rate began to increase at an LVEF of 55% to 60%, with a survival rate similar to that of women with LVEF less than or equal to 55% (Figure 1B). Similarly, men with the lowest LV-GLS (<19.9%) demonstrated the worst survival, with similar survival rates observed among the other groups (Figure 1C). In women, there was a stepwise increase in mortality across LV-GLS groups (Figure 1D). These trends were generally consistent when stratified by symptom status (eTable 2 in Supplement 1). Furthermore, the sensitivity analysis showed consistent results across different time periods, except for the association involving LV-GLS in women in the later period (years 2015-2020; eFigure 4 in Supplement 1).

In the univariable and multivariable Cox analyses, decreased LVEF (as a continuous variable) was significantly associated with a higher risk of mortality in the entire cohort and in both men and women (Table 2). Regarding the LVEF categories, the univariable analysis showed that compared with those with an LVEF greater than 60%, women with an LVEF of 55% to 60% and less than or equal to 55% had higher risks of mortality. In men, however, only an LVEF less than or equal to 55% was associated with a higher risk of mortality. After multivariable adjustment, this association remained significant only in women (LVEF ≤55%, adjusted HR, 3.48; 95% CI, 1.84-6.58; *P* < .001; LVEF 55%-60%, adjusted HR, 2.21; 95% CI, 1.36-3.58; *P* = .001), with a significant interaction by sex (*P *for interaction by sex = .02).

**Table 2.** Association of Left Ventricular Systolic Function With Risk of Mortality After Surgery According to Sex

Variable	All patients (N = 1686)		Men (n = 1088)		Women (n = 598)		*P* value for interaction by sex
	HR (95% CI)	*P* value	HR (95% CI)	*P* value	HR (95% CI)	*P* value	
LVEF, per 1% decrease							
Univariable analysis	1.05 (1.03-1.06)	<.001	1.04 (1.02-1.07)	<.001	1.05 (1.03-1.07)	<.001	.69
Multivariable analysis^a^	1.05 (1.03-1.07)	<.001	1.03 (1.00-1.06)	.04	1.06 (1.03-1.09)	<.001	.14
LVEF groups							
Univariable analysis							
LVEF >60%	1 [Reference]	NA	1 [Reference]	NA	1 [Reference]	NA	.09
LVEF 55%-60%	1.67 (1.20-2.33)	.002	1.26 (0.78-2.02)	.35	2.56 (1.61-4.09)	<.001	
LVEF ≤55%	2.59 (1.86-3.62)	<.001	2.30 (1.47-3.60)	<.001	3.46 (2.09-5.72)	<.001	
Multivariable analysis^a^							
LVEF >60%	1 [Reference]	NA	1 [Reference]	NA	1 [Reference]	NA	.02
LVEF 55%-60%	1.45 (1.02-2.06)	.03	0.97 (0.58-1.61)	.90	2.21 (1.36-3.58)	.001	
LVEF ≤55%	2.33 (1.54-3.54)	<.001	1.58 (0.90-2.77)	.11	3.48 (1.84-6.58)	<.001	
LV-GLS, per 1% decrease							
Univariable analysis	1.09 (1.06-1.12)	<.001	1.08 (1.04-1.12)	<.001	1.12 (1.07-1.16)	<.001	.33
Multivariable analysis^a^	1.05 (1.01-1.08)	.008	1.02 (0.97-1.07)	.41	1.07 (1.02-1.13)	.005	.23
LV-GLS groups							
Univariable analysis							
LV-GLS ≥23.4%	1 [Reference]	NA	1 [Reference]	NA	1 [Reference]	NA	.54
LV-GLS 19.9%-23.4%	1.22 (0.85-1.76)	.28	1.07 (0.65-1.77)	.79	1.58 (0.93-2.68)	.09	
LV-GLS <19.9%	2.38 (1.71-3.29)	<.001	2.09 (1.33-3.27)	.001	3.03 (1.88-4.88)	<.001	
Multivariable analysis^a^							
LV-GLS ≥23.4%	1 [Reference]	NA	1 [Reference]	NA	1 [Reference]	NA	.31
LV-GLS 19.9%-23.4%	1.00 (0.68-1.45)	.98	0.77 (0.45-1.29)	.32	1.29 (0.75-2.22)	.35	
LV-GLS <19.9%	1.51 (1.05-2.18)	.02	1.14 (0.68-1.92)	.61	1.96 (1.17-3.28)	.01	

Abbreviations: HR, hazard ratio; LVEF, left ventricular ejection fraction; LV-GLS, left ventricular global longitudinal strain; NA, not applicable.

^a^ Adjusted for age, sex, symptomatic mitral regurgitation, atrial fibrillation, left atrial dimension, tricuspid regurgitation peak velocity, and left ventricular end-systolic dimension.

For LV-GLS, decreased and lower LV-GLS categories were associated with increased mortality risks in the univariable analysis, irrespective of sex. After multivariable adjustment, this association remained significant in women but showed no significant interaction by sex (Table 2).

**Figure 2.**
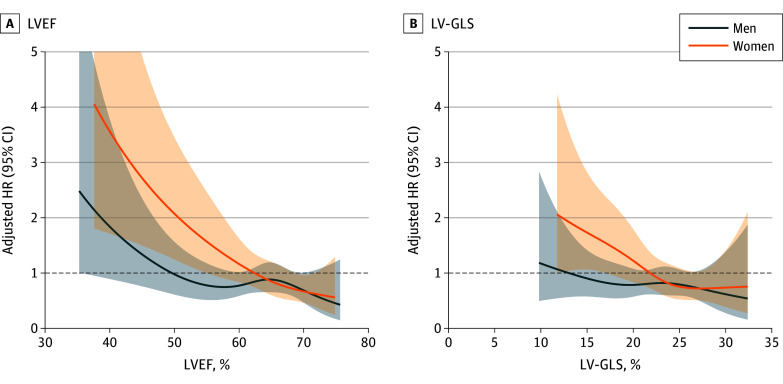
Association of Left Ventricular Systolic Function With Mortality Risk by Sex Restricted cubic spline curves illustrate the associations of left ventricular ejection fraction (LVEF) and left ventricular global longitudinal strain (LV-GLS) with mortality among men and women. The hazard ratios (HRs) are adjusted for age, symptoms, atrial fibrillation, left atrial dimension, tricuspid regurgitation peak velocity, and left ventricular end-systolic dimension. Shaded areas denote 95% CIs. Dashed lines denote the null value (HR = 1).

The RCS curves for LVEF and LV-GLS by sex are shown in Figure 2. Adjusted mortality risk increased with decreasing LVEF in both sexes; however, the pattern varied by sex. In men, the risk began to increase at LVEF levels between approximately 50% to 60%, whereas in women, the increase was gradual and started at higher LVEF levels (>60%). For LV-GLS, mortality risk increased below an LV-GLS of 20% to 25% in women, but no clear threshold was evident for men.

### Sensitivity Analysis on the Propensity-Matched Cohort

In the propensity-matched cohort, men and women had similar age and prevalence of symptoms and received similar interventions (eTable 3 in Supplement 1). Overall survival was comparable between sexes in this matched cohort (eFigure 3 in Supplement 1). Similar to the original cohort, men with the lowest LVEF (≤55%) and LV-GLS (<19.9%) had the worst survival, with similar survival rates among the other groups, whereas women exhibited an earlier increase in mortality (eFigure 5 in Supplement 1).

### Subgroup Analysis in Asymptomatic Patients

**Figure 3.**
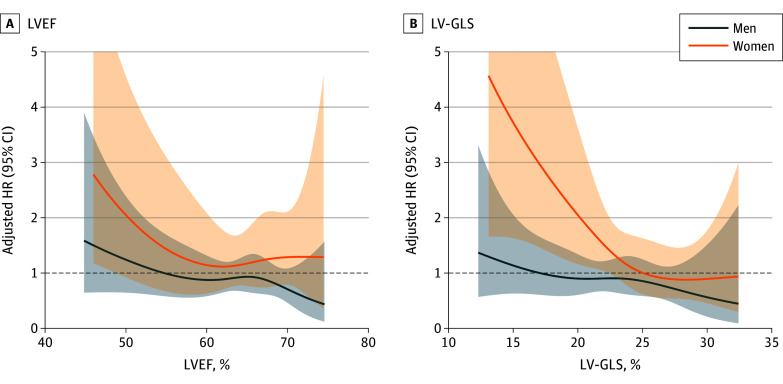
Association of Left Ventricular Systolic Function With Mortality Risk by Sex in Asymptomatic Individuals Restricted cubic spline curves illustrate the associations of left ventricular ejection fraction (LVEF) and left ventricular global longitudinal strain (LV-GLS) with mortality, among asymptomatic men and women. The hazard ratios (HRs) are adjusted for age, atrial fibrillation, left atrial dimension, tricuspid regurgitation peak velocity, and left ventricular end-systolic dimension. Shaded areas denote 95% CIs. Dashed lines denote the null value (HR = 1).

Of 1686 individuals in the entire population, 1050 (718 men [68.4%] and 332 women [31.6%]) were asymptomatic. Similar to the entire cohort, women were older and had higher LVEF and LV-GLS than did men (eTable 4 in Supplement 1). In the Kaplan-Meier survival curves, men with LVEF less than or equal to 55% or LV-GLS less than 19.9% exhibited higher mortality, whereas in women, an increase in mortality was observed with higher LVEF and LV-GLS categories (eFigure 6 in Supplement 1). RCS curves demonstrated clear inflection points for LVEF and LV-GLS among women, whereas no distinct threshold was observed in men (Figure 3). Among asymptomatic patients without evidence of obstructive coronary artery disease, a prominent threshold for LV-GLS was noted in women, but not in men (eFigure 7 in Supplement 1).

## Discussion

This cohort study, which used a large strain imaging database, found a sex-specific association between LV systolic function and long-term mortality in patients with severe degenerative MR. Women exhibited significantly better LV systolic function, as indicated by LVEF and LV-GLS. A decline in LVEF and LV-GLS was associated with higher mortality risk in both sexes; however, the pattern of these associations varied by sex. Women had an earlier increase in mortality risk at higher levels of LVEF and LV-GLS. These findings remained consistent when analyzing asymptomatic patients separately. This study highlights the importance of considering sex differences in the prognostic value of LV systolic dysfunction and emphasizes the potential need for sex-specific criteria to optimize the timing of intervention in patients with severe degenerative MR.

Data demonstrating sex differences in MR are limited. A previous study^16^ has shown that, among patients with severe degenerative MR, women were older, had smaller chamber sizes, and had better LV systolic function than men, a trend observed in our cohort. Surgical outcomes also differed by sex; women with severe MR were more likely to be referred for surgery later and experienced more adverse events compared with men.^16,17,18^ Therefore, the identification of sex differences in risk factors and their prognostic importance and integrating these insights into the decision-making process for intervention may improve survival for patients with severe degenerative MR.

Current guidelines indicate that an LVEF of less than or equal to 60% serves as a marker of LV decompensation, which is an indication for surgical intervention in asymptomatic patients.^19,20^ However, this cutoff value has not been validated through clinical trials but is based on a few observational studies.^21^ It remains unclear whether the association between LV systolic function and long-term prognosis differs by sex, or whether the same cutoff values for LV decompensation apply to both sexes. A recent study^11^ showed that women exhibit an earlier increase in mortality risk at lower LV end-systolic dimension compared with men, even after adjusting for body surface area, and have a higher mortality risk with decreasing LVEF. In our study, we showed that the association between LVEF and long-term mortality, as illustrated by RCS and Kaplan-Meier curves, varies by sex: in women, the mortality risk appeared to increase gradually, starting from higher LVEF levels. Our findings suggest early surgery for women with an LVEF greater than 60%, as watchful waiting until their LVEF falls below 60% may lead to worse outcomes.

Sex differences were also observed in the prognostic impact of LV-GLS, with a more pronounced increase in mortality risk associated with a decrease in LV-GLS noted for women. LV-GLS is an established marker of LV decompensation in MR, offering incremental prognostic value beyond the conventional LVEF.^6,8^ Previous studies have suggested that the threshold for LV-GLS to discriminate the risk of mortality or long-term LV dysfunction in severe MR ranges between 18% and 21%.^6,7^ Our study identified sex-specific variations in this association, suggesting potentially higher cutoff values for women compared with men in the RCS curves. Moreover, a lower LV-GLS was associated with increased mortality risk, even when asymptomatic patients were considered separately. LV-GLS may improve predictive performance beyond LVEF, as it reflects longitudinal fiber shortening rather than changes in chamber volume.^22,23^

The exact mechanism underlying the more pronounced mortality risk associated with LV systolic dysfunction in women remains unclear. This may be attributable to unmeasured confounders, such as a different propensity for symptom development, frailty, or delayed referral.^24^ Although there was a significant age difference between sexes in our cohort, similar findings were observed in the propensity-matched analysis, emphasizing the importance of sex-specific considerations in surgical decision-making. Moreover, female sex is considered a risk factor in the Society of Thoracic Surgeons’ score for surgical MV replacement.^24,25^ This highlights the need for more meticulous management and postsurgical care for women following MV surgery.

Treatment options for severe MR are expanding, including early intervention in asymptomatic patients^26^ and transcatheter edge-to-edge repair.^27,28^ Risk stratification and adverse event estimation in patients with severe MR are imperative to implement a more tailored treatment strategy.^29,30^ Future studies should explore the optimal timing and methods of intervention in severe MR, focusing on the sex-specific association of LV systolic dysfunction with adverse events and investigating the potential of LV-GLS as a novel marker for these assessments.

### Limitations

This study has limitations that should be mentioned. First, although there was a trend suggesting that the relationship between LV-GLS and mortality may vary by sex, this association did not reach statistical significance for a sex interaction. Larger studies with sufficient power to confirm sex differences and establish sex-specific thresholds for LV-GLS are warranted. Second, more detailed clinical end points, such as cardiovascular death, heart failure, or all-cause hospitalization, were unavailable. Hospitalization outcomes may encompass various reasons, including admissions for elective procedures, and the adjudication of these events may be unreliable in this retrospective setting. In contrast, all-cause mortality provides the most unbiased study outcome, commonly used in multiple studies, including those involving degenerative MR.^1,29,30^ Third, we could not incorporate postsurgical information into our analysis, including surgical complications, residual MR, recurrent MR, or prosthetic valve dysfunction. Fourth, our study cannot elucidate the mechanisms underlying these sex disparities.

## Conclusions

In this cohort study of patients with severe degenerative MR, decreased LVEF and LV-GLS were associated with higher long-term postsurgical mortality in both sexes. Women had an earlier increase in mortality at higher levels of LVEF and LV-GLS compared with men. These findings suggest a potential need to establish sex-specific criteria for LV decompensation to guide the timing of early surgery in severe degenerative MR.

## Notes

Supplement 1. **eMethods**. Echocardiography measurements **eTable 1.** Number of mortality events in men and women by different time periods **eTable 2.** Number of patients and mortality events according to symptom status, left ventricular ejection fraction, and left ventricular global longitudinal strain groups by sex **eTable 3.** Baseline characteristics of propensity-matched cohort by sex **eTable 4.** Baseline characteristics of asymptomatic study patients by sex **eFigure 1.** Proportion of missing values for the main study variables **eFigure 2.** Distribution of left ventricular ejection fraction and left ventricular global longitudinal strain values according to sex **eFigure 3.** Mortality according to sex in the entire cohort and the propensity-matched cohort **eFigure 4.** Mortality according to left ventricular systolic function and sex stratified by the different time period **eFigure 5.** Mortality according to left ventricular systolic function and sex in the propensity-matched cohort **eFigure 6.** Mortality according to left ventricular systolic function and sex in asymptomatic individuals **eFigure 7.** Relationship between left ventricular systolic function and mortality by sex in asymptomatic individuals without evidence of obstructive coronary artery disease

Supplement 2.
Data Sharing Statement

## References

[1] Grigioni F, Clavel MA, Vanoverschelde JL, ; MIDA Investigators. The MIDA Mortality Risk Score: development and external validation of a prognostic model for early and late death in degenerative mitral regurgitation. Eur Heart J. 2018;39(15):1281-1291. doi:10.1093/eurheartj/ehx465 29020352

[2] Antoine C, Benfari G, Michelena HI, . Clinical outcome of degenerative mitral regurgitation: critical importance of echocardiographic quantitative assessment in routine practice. Circulation. 2018;138(13):1317-1326. doi:10.1161/CIRCULATIONAHA.117.033173 29853518

[3] Badhwar V, Vemulapalli S, Mack MA, . Volume-outcome association of mitral valve surgery in the United States. JAMA Cardiol. 2020;5(10):1092-1101. doi:10.1001/jamacardio.2020.2221 32609292 PMC7330833

[4] Gaasch WH, Meyer TE. Left ventricular response to mitral regurgitation: implications for management. Circulation. 2008;118(22):2298-2303. doi:10.1161/CIRCULATIONAHA.107.755942 19029478

[5] Smiseth OA, Torp H, Opdahl A, Haugaa KH, Urheim S. Myocardial strain imaging: how useful is it in clinical decision making? Eur Heart J. 2016;37(15):1196-1207. doi:10.1093/eurheartj/ehv529 26508168 PMC4830908

[6] Hiemstra YL, Tomsic A, van Wijngaarden SE, . Prognostic value of global longitudinal strain and etiology after surgery for primary mitral regurgitation. JACC Cardiovasc Imaging. 2020;13(2 Pt 2):577-585. doi:10.1016/j.jcmg.2019.03.024 31202761

[7] Witkowski TG, Thomas JD, Debonnaire PJ, . Global longitudinal strain predicts left ventricular dysfunction after mitral valve repair. Eur Heart J Cardiovasc Imaging. 2013;14(1):69-76. doi:10.1093/ehjci/jes155 22848021

[8] Kim HM, Cho GY, Hwang IC, . Myocardial strain in prediction of outcomes after surgery for severe mitral regurgitation. JACC Cardiovasc Imaging. 2018;11(9):1235-1244. doi:10.1016/j.jcmg.2018.03.016 29778855

[9] Alashi A, Mentias A, Patel K, . Synergistic utility of brain natriuretic peptide and left ventricular global longitudinal strain in asymptomatic patients with significant primary mitral regurgitation and preserved systolic function undergoing mitral valve surgery. Circ Cardiovasc Imaging. 2016;9(7):e004451. doi:10.1161/CIRCIMAGING.115.004451 27342145

[10] Mentias A, Naji P, Gillinov AM, . Strain echocardiography and functional capacity in asymptomatic primary mitral regurgitation with preserved ejection fraction. J Am Coll Cardiol. 2016;68(18):1974-1986. doi:10.1016/j.jacc.2016.08.030 27591831

[11] Abadie BQ, Cremer PC, Vakamudi S, Gillinov AM, Svensson LG, Cho L. Sex-specific prognosis of left ventricular size and function following repair of degenerative mitral regurgitation. J Am Coll Cardiol. 2024;83(2):303-312. doi:10.1016/j.jacc.2023.10.033 38199708

[12] World Medical Association. World Medical Association Declaration of Helsinki: ethical principles for medical research involving human subjects. JAMA. 2013;310(20):2191-2194. doi:10.1001/jama.2013.281053 24141714

[13] Lang RM, Badano LP, Mor-Avi V, . Recommendations for cardiac chamber quantification by echocardiography in adults: an update from the American Society of Echocardiography and the European Association of Cardiovascular Imaging. J Am Soc Echocardiogr. 2015;28(1):1-39.e14. doi:10.1016/j.echo.2014.10.003 25559473

[14] Zoghbi WA, Adams D, Bonow RO, . Recommendations for noninvasive evaluation of native valvular regurgitation: a report from the American Society of Echocardiography developed in collaboration with the Society for Cardiovascular Magnetic Resonance. J Am Soc Echocardiogr. 2017;30(4):303-371. doi:10.1016/j.echo.2017.01.007 28314623

[15] R Core Team. R: a language and environment for statistical computing. 2023. Accessed February 19, 2025. https://www.R-project.org/

[16] Avierinos JF, Inamo J, Grigioni F, Gersh B, Shub C, Enriquez-Sarano M. Sex differences in morphology and outcomes of mitral valve prolapse. Ann Intern Med. 2008;149(11):787-795. doi:10.7326/0003-4819-149-11-200812020-00003 19047025 PMC2897166

[17] Mantovani F, Clavel MA, Michelena HI, Suri RM, Schaff HV, Enriquez-Sarano M. Comprehensive imaging in women with organic mitral regurgitation: implications for clinical outcome. JACC Cardiovasc Imaging. 2016;9(4):388-396. doi:10.1016/j.jcmg.2016.02.017 27056158

[18] Liu K, Ye Q, Zhao Y, Zhao C, Song L, Wang J. Sex differences in the outcomes of degenerative mitral valve repair. Ann Thorac Cardiovasc Surg. 2023;29(4):192-199. doi:10.5761/atcs.oa.22-00210 36908120 PMC10466113

[19] Otto CM, Nishimura RA, Bonow RO, . 2020 ACC/AHA guideline for the management of patients with valvular heart disease: a report of the American College of Cardiology/American Heart Association Joint Committee on Clinical Practice Guidelines. Circulation. 2021;143(5):e72-e227. doi:10.1161/CIR.0000000000000923 33332150

[20] Vahanian A, Beyersdorf F, Praz F, ; ESC/EACTS Scientific Document Group. 2021 ESC/EACTS Guidelines for the management of valvular heart disease. Eur Heart J. 2022;43(7):561-632. doi:10.1093/eurheartj/ehab395 34453165

[21] Enriquez-Sarano M, Tajik AJ, Schaff HV, Orszulak TA, Bailey KR, Frye RL. Echocardiographic prediction of survival after surgical correction of organic mitral regurgitation. Circulation. 1994;90(2):830-837. doi:10.1161/01.CIR.90.2.830 8044955

[22] Namazi F, van der Bijl P, Hirasawa K, . Prognostic value of left ventricular global longitudinal strain in patients with secondary mitral regurgitation. J Am Coll Cardiol. 2020;75(7):750-758. doi:10.1016/j.jacc.2019.12.024 32081284

[23] Tops LF, Delgado V, Marsan NA, Bax JJ. Myocardial strain to detect subtle left ventricular systolic dysfunction. Eur J Heart Fail. 2017;19(3):307-313. doi:10.1002/ejhf.694 27891719

[24] DesJardin JT, Chikwe J, Hahn RT, Hung JW, Delling FN. Sex differences and similarities in valvular heart disease. Circ Res. 2022;130(4):455-473. doi:10.1161/CIRCRESAHA.121.319914 35175844 PMC8869851

[25] O’Brien SM, Feng L, He X, . The Society of Thoracic Surgeons 2018 adult cardiac surgery risk models: part 2—statistical methods and results. Ann Thorac Surg. 2018;105(5):1419-1428. doi:10.1016/j.athoracsur.2018.03.003 29577924

[26] Kang DH, Park SJ, Sun BJ, . Early surgery versus conventional treatment for asymptomatic severe mitral regurgitation: a propensity analysis. J Am Coll Cardiol. 2014;63(22):2398-2407. doi:10.1016/j.jacc.2014.02.577 24694528

[27] Feldman T, Kar S, Elmariah S, ; EVEREST II Investigators. Randomized comparison of percutaneous repair and surgery for mitral regurgitation: 5-year results of EVEREST II. J Am Coll Cardiol. 2015;66(25):2844-2854. doi:10.1016/j.jacc.2015.10.018 26718672

[28] Feldman T, Foster E, Glower DD, ; EVEREST II Investigators. Percutaneous repair or surgery for mitral regurgitation. N Engl J Med. 2011;364(15):1395-1406. doi:10.1056/NEJMoa1009355 21463154

[29] Kwak S, Lee SA, Lim J, . Long-term outcomes in distinct phenogroups of patients with primary mitral regurgitation undergoing valve surgery. Heart. 2023;109(4):305-313. doi:10.1136/heartjnl-2022-321305 35882521 PMC9887360

[30] Kwak S, Lee SA, Lim J, . Data-driven mortality risk prediction of severe degenerative mitral regurgitation patients undergoing mitral valve surgery. Eur Heart J Cardiovasc Imaging. 2023;24(9):1156-1165. doi:10.1093/ehjci/jead077 37115641

