# Supplementary material for: Sex-Specific Association of Left Ventricular Function With Mortality in Severe Mitral Regurgitation
Source: JAMA Netw Open. 2025 Mar 31;8(3):e252420. doi: 10.1001/jamanetworkopen.2025.2420 (PMC11959442; doi:10.1001/jamanetworkopen.2025.2420)
Supplement: Supplement 1. — eMethods. Echocardiography measurements eTable 1. Number of mortality events in men and women by different time periods eTable 2. Number of patients and mortality events according to symptom status, left ventricular ejection fraction, and left ventricular global longitudinal strain groups by sex eTable 3. Baseline characteristics of propensity-matched cohort by sex eTable 4. Baseline characteristics of asymptomatic study patients by sex eFigure 1. Proportion of missing values for the main study variables eFigure 2. Distribution of left ventricular ejection fraction and left ventricular global longitudinal strain values according to sex eFigure 3. Mortality according to sex in the entire cohort and the propensity-matched cohort eFigure 4. Mortality according to left ventricular systolic function and sex stratified by the different time period eFigure 5. Mortality according to left ventricular systolic function and sex in the propensity-matched cohort eFigure 6. Mortality according to left ventricular systolic function and sex in asymptomatic individuals eFigure 7. Relationship between left ventricular systolic function and mortality by sex in asymptomatic individuals without evidence of obstructive coronary artery disease [file jamanetwopen-e252420-s001.pdf]

# Supplemental Online Content

Kwak S, Sun BJ, Lee S, et al. Sex-specific association of left ventricular function with mortality in severe mitral regurgitation. *JAMA Netw Open*. 2025;8(3):e252420. doi:10.1001/jamanetworkopen.2025.2420

**eMethods.** Echocardiography measurements
**eTable 1.** Number of mortality events in men and women by different time periods
**eTable 2.** Number of patients and mortality events according to symptom status, left ventricular ejection fraction, and left ventricular global longitudinal strain groups by sex
**eTable 3.** Baseline characteristics of propensity-matched cohort by sex
**eTable 4.** Baseline characteristics of asymptomatic study patients by sex
**eFigure 1.** Proportion of missing values for the main study variables
**eFigure 2.** Distribution of left ventricular ejection fraction and left ventricular global longitudinal strain values according to sex
**eFigure 3.** Mortality according to sex in the entire cohort and the propensity-matched cohort
**eFigure 4.** Mortality according to left ventricular systolic function and sex stratified by the different time period
**eFigure 5.** Mortality according to left ventricular systolic function and sex in the propensity-matched cohort
**eFigure 6.** Mortality according to left ventricular systolic function and sex in asymptomatic individuals
**eFigure 7.** Relationship between left ventricular systolic function and mortality by sex in asymptomatic individuals without evidence of obstructive coronary artery disease

This supplemental material has been provided by the authors to give readers additional information about their work.

© 2025 Kwak S et al. *JAMA Network Open*.

**eMethods. Echocardiography measurements**

LV dimensions were measured from the parasternal view, perpendicular to the LV long axis. LV end-diastolic and end-systolic volumes were measured by tracing the endocardial border in the apical 2-chamber and 4-chamber views. LV ejection fraction was calculated using the biplane Simpson's method. The LV mass was calculated according to the recommended formula and indexed to the body surface area. The anteroposterior dimension of the left atrium (LA) was measured at the end of systole in the standard parasternal views. The early diastolic velocity of the mitral inflow (E-wave velocity) was acquired from the apical 4-chamber view at the tip of the MV. The septal e'-wave was obtained at the medial side of the mitral annulus. Tricuspid regurgitation (TR) peak velocity was measured from the right ventricle-focused apical 4-chamber view. The severity of MR was determined using qualitative, semiquantitative, and quantitative methods following contemporary guidelines.

© 2025 Kwak S et al. *JAMA Network Open*.

**eTable 1. Number of mortality events in men and women by different time periods**

| | Men (n=1,088) | Women (n=598) | Log-rank P |
|---|---|---|---|
| **30-day mortality** | 7 (0.6) | 3 (0.5) | 0.72 |
| **1-year mortality** | 28 (2.6) | 20 (3.3) | 0.37 |
| **5-year mortality** | 59 (5.4) | 43 (7.2) | 0.15 |
| **8-year mortality** | 78 (7.2) | 66 (11.0) | 0.01 |
| **Overall mortality** | 117 (10.8) | 103 (17.2) | <0.001 |

© 2025 Kwak S et al. *JAMA Network Open*.

**eTable 2. Number of patients and mortality events according to symptom status, left ventricular ejection fraction, and left ventricular global longitudinal strain groups by sex**

| | **Men (n=1,088)** | | **Women (n=598)** | |
|---|---|---|---|---|
| | **Asymptomatic (n=718)** | **Symptomatic (n=370)** | **Asymptomatic (n=332)** | **Symptomatic (n=266)** |
| **LVEF >60%** | n=535 (39 deaths [7.3%]) | n=218 (28 deaths [12.8%]) | n=264 (24 deaths [9.1%]) | n=189 (32 deaths [16.9%]) |
| **LVEF 55−60%** | n=121 (12 deaths [9.9%]) | n=81 (11 deaths [13.6%]) | n=42 (7 deaths [16.7%]) | n=46 (19 deaths [41.3%]) |
| **LVEF ≤55%** | n=62 (12 deaths [19.4%]) | n=71 (15 deaths [21.1%]) | n=25 (6 deaths [24.0%]) | n=31 (15 deaths [48.4%]) |
| **LV-GLS ≥23.4%** | n=269 (19 deaths [7.1%]) | n=71 (10 deaths [14.1%]) | n=152 (11 deaths [7.2%]) | n=81 (16 deaths [19.8%]) |
| **LV-GLS 19.9−23.4%** | n=229 (15 deaths [6.6%]) | n=136 (17 deaths [12.5%]) | n=97 (12 deaths [12.4%]) | n=90 (16 deaths [17.8%]) |
| **LV-GLS <19.9%** | n=220 (29 deaths [13.2%]) | n=163 (27 deaths [16.6%]) | n=83 (14 deaths [16.9%]) | n=95 (34 deaths [35.8%]) |

There was one female patient with missing LVEF data who was omitted from the LVEF categorization.

LVEF, left ventricular ejection fraction; LV-GLS, left ventricular global longitudinal strain

© 2025 Kwak S et al. *JAMA Network Open*.

**eTable 3. Baseline characteristics of propensity-matched cohort by sex**

| Characteristics | Men (n=578) | Women (n=578) | P |
|---|---|---|---|
| Age, years | 60 (51−68) | 62 (51−69) | 0.207 |
| Body mass index, kg/m$^2$ | 24.4 (22.4−26.4) | 23.8 (21.4−26.3) | 0.005 |
| Comorbidities | | | |
| Hypertension | 250 (43.3) | 244 (42.2) | 0.766 |
| Diabetes mellitus | 71 (12.3) | 53 (9.2) | 0.106 |
| Atrial fibrillation | 243 (42.0) | 185 (32.0) | 0.001 |
| Stroke | 13 (2.2) | 23 (4.0) | 0.128 |
| Myocardial infarction | 9 (1.6) | 10 (1.7) | >0.99 |
| Symptomatic MR | 252 (43.6) | 247 (42.7) | 0.812 |
| Symptoms | | | |
| Dyspnea | 218 (37.7) | 219 (37.9) | >0.99 |
| Chest pain | 32 (5.5) | 19 (3.3) | 0.086 |
| Edema | 23 (4.0) | 22 (3.8) | >0.99 |
| Palpitation | 39 (6.7) | 41 (7.1) | 0.908 |
| Syncope | 4 (0.7) | 8 (1.4) | 0.384 |
| Laboratory results | | | |
| Hemoglobin, g/dL | 14.1 (13.0−14.9) | 12.5 (11.5−13.2) | <0.001 |
| eGFR, mL/min/1.73m$^2$ | 84.5 (71.0−94.8) | 86.4 (67.3−98.7) | 0.340 |
| MR surgery type | | | 0.145 |
| MV repair | 517 (89.4) | 507 (87.7) | |
| MV replacement (mechanical) | 26 (4.5) | 20 (3.5) | |
| MV replacement (bioprosthetic) | 35 (6.1) | 51 (8.8) | |
| Concomitant CABG | 40 (6.9) | 29 (5.0) | 0.214 |
| Concomitant surgical atrial ablation | 213 (36.9) | 168 (29.1) | 0.006 |
| **Echocardiography** | | | |
| LV end-systolic diameter, mm | 38 (34−42) | 35 (32−39) | <0.001 |
| LV end-diastolic diameter, mm | 61 (57−65) | 57 (53−62) | <0.001 |
| LV end-systolic volume, mL | 59.0 (48.0−74.0) | 46.0 (36.0−59.0) | <0.001 |
| LV end-systolic volume index, mL/m$^2$ | 32.8 (26.7−41.0) | 28.9 (22.5−37.8) | <0.001 |
| LV end-diastolic volume, mL | 167.0 (137.0−196.5) | 133.0 (110.0−163.0) | <0.001 |

© 2025 Kwak S et al. *JAMA Network Open.*

| | | | |
|---|---|---|---|
| LV end-diastolic volume index, mL/$m^2$ | 92.2 (75.5−109.6) | 85.6 (68.6−103.4) | <0.001 |
| LVEF, % | 63.0 (59.3−67.2) | 64.6 (60.2−68.6) | <0.001 |
| LV mass index, g/$m^2$ | 136.9 (119.5−156.8) | 128.2 (108.6−152.1) | <0.001 |
| LA dimension, mm | 52 (47−59) | 50 (44−56) | <0.001 |
| E-wave, m/s | 1.14 (0.82−1.41) | 1.16 (0.75−1.43) | 0.670 |
| e'-wave, cm/s | 7.3 (6.0− 9.0) | 7.0 (5.8− 9.0) | 0.011 |
| E/e' ratio | 15 (12−20) | 17 (13−22) | 0.002 |
| TR peak velocity, m/s | 2.8 (2.5− 3.3) | 2.9 (2.6− 3.4) | 0.001 |
| LV-GLS, % | 20.9 (18.3−23.7) | 22.1 (19.1−25.1) | <0.001 |

Values are expressed as median (interquartile range) or numbers (percentage)

CABG, coronary artery bypass grafting; eGFR, estimated glomerular filtration rate; LA, left atrial; LV, left ventricular; LVEF, left ventricular ejection fraction; LV-GLS, left ventricular global longitudinal strain; MR, mitral regurgitation; MV, mitral valve; TR, tricuspid regurgitation

**eTable 4. Baseline characteristics of asymptomatic study patients by sex**

| **Characteristics** | **Men (n=718)** | **Women (n=332)** | **P** |
|---|---|---|---|
| Age, years | 54 (45–62) | 60 (50–69) | <0.001 |
| Body mass index, kg/m² | 24.8 (22.8–26.9) | 23.5 (21.6–26.3) | <0.001 |
| Comorbidities | | | |
| Hypertension | 292 (40.7) | 142 (42.8) | 0.565 |
| Diabetes mellitus | 61 (8.5) | 25 (7.5) | 0.682 |
| Atrial fibrillation | 220 (30.6) | 99 (29.8) | 0.844 |
| Stroke | 15 (2.1) | 15 (4.5) | 0.046 |
| Myocardial infarction | 17 (2.4) | 6 (1.8) | 0.726 |
| Laboratory results | | | |
| Hemoglobin, g/dL | 14.2 (13.4–15.0) | 12.4 (11.5–13.2) | <0.001 |
| eGFR, mL/min/1.73m² | 89.7 (77.0–101.0) | 89.0 (72.0–100.4) | 0.455 |
| MR surgery type | | | <0.001 |
| MV repair | 678 (94.4) | 300 (90.4) | |
| MV replacement (mechanical) | 29 (4.0) | 8 (2.4) | |
| MV replacement (bioprosthetic) | 11 (1.5) | 24 (7.2) | |
| Concomitant CABG | 28 (3.9) | 13 (3.9) | >0.99 |
| Concomitant surgical atrial ablation | 199 (27.7) | 90 (27.1) | 0.896 |
| **Echocardiography** | | | |
| LV end-systolic diameter, mm | 38 (35–42) | 35 (32–39) | <0.001 |
| LV end-diastolic diameter, mm | 61 (57–65) | 57 (53–62) | <0.001 |
| LV end-systolic volume, mL | 60.0 (50.0–73.0) | 46.0 (37.0–57.0) | <0.001 |
| LV end-systolic volume index, mL/m² | 32.3 (26.9–39.6) | 29.0 (23.1–37.1) | <0.001 |
| LV end-diastolic volume, mL | 170.0 (141.5–201.0) | 135.0 (110.0–160.0) | <0.001 |
| LV end-diastolic volume index, mL/m² | 91.5 (76.6–109.5) | 86.7 (68.6–101.4) | <0.001 |
| LVEF, % | 64.0 (60.0–67.4) | 64.8 (60.9–69.0) | 0.003 |
| LV mass index, g/m² | 133.2 (114.4–152.7) | 127.0 (107.3–150.3) | 0.002 |
| LA dimension, mm | 50.0 (45.0–56.0) | 48.0 (44.0–54.0) | 0.018 |
| E-wave, m/s | 1.11 (0.88–1.38) | 1.18 (0.91–1.43) | 0.034 |
| e’-wave, cm/s | 8.1 (6.7–9.9) | 7.2 (5.9–9.0) | <0.001 |
| E/e’ ratio | 13 (10–18) | 16 (13–20) | <0.001 |

© 2025 Kwak S et al. *JAMA Network Open*.

| | | | |
|---|---|---|---|
| TR peak velocity, m/s | 2.6 (2.4−3.0) | 2.8 (2.5−3.3) | <0.001 |
| LV-GLS, % | 22.0 (19.0−24.8) | 22.9 (19.9−25.5) | 0.008 |

Values are expressed as median (interquartile range) or numbers (percentage)

CABG, coronary artery bypass grafting; eGFR, estimated glomerular filtration rate; LA, left atrial; LV, left ventricular; LVEF, left ventricular ejection fraction; LV-GLS, left ventricular global longitudinal strain; MV, mitral valve; TR, tricuspid regurgitation

© 2025 Kwak S et al. *JAMA Network Open*.

**eFigure 1. Proportion of missing values for the main study variables**

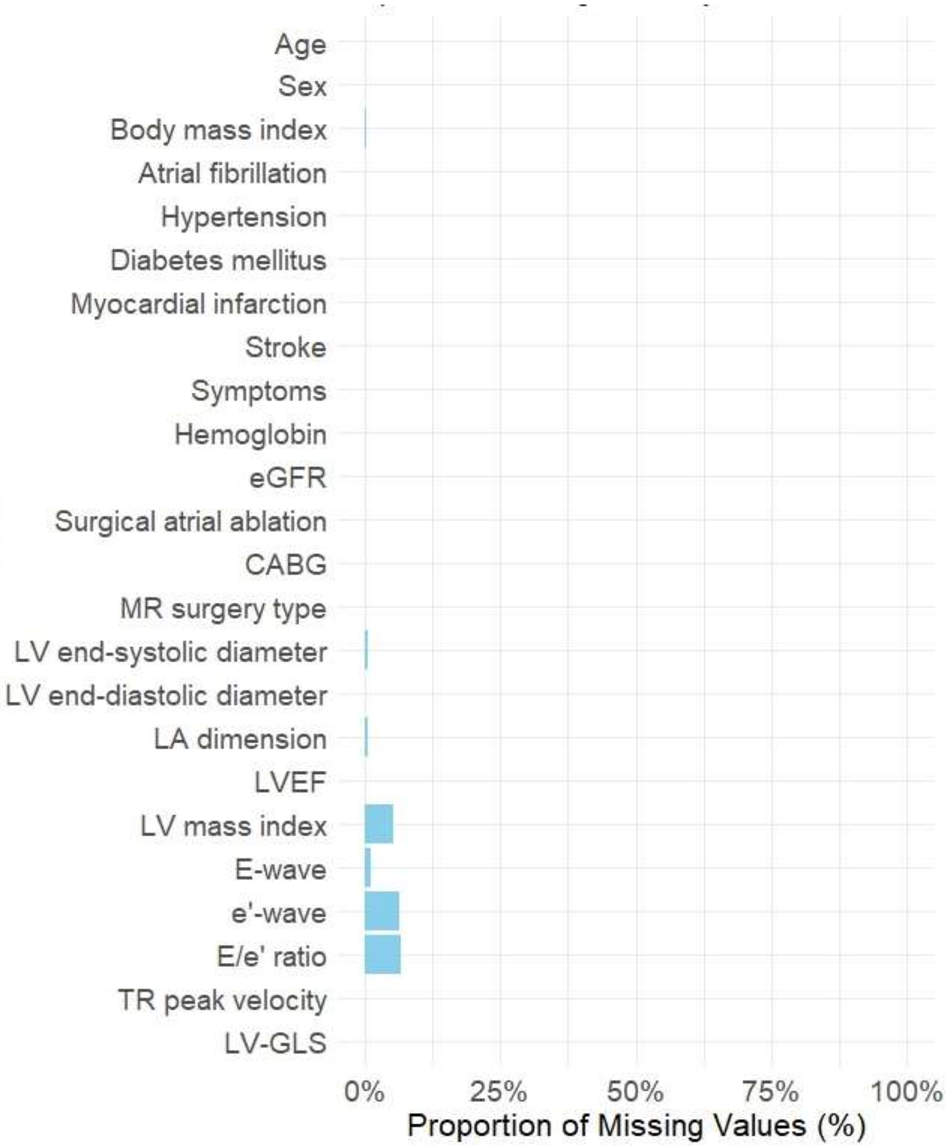


CABG, coronary artery bypass grafting; eGFR, estimated glomerular filtration rate; LA, left atrial; LV, left ventricle; LVEF, left ventricular ejection fraction; LV-GLS, left ventricular global longitudinal strain; MR, mitral regurgitation; TR, tricuspid regurgitation.

© 2025 Kwak S et al. *JAMA Network Open*.

**eFigure 2. Distribution of left ventricular ejection fraction and left ventricular global longitudinal strain values according to sex**

For LVEF, the data are grouped in intervals of 2%, and for LV-GLS, the intervals are 1%.

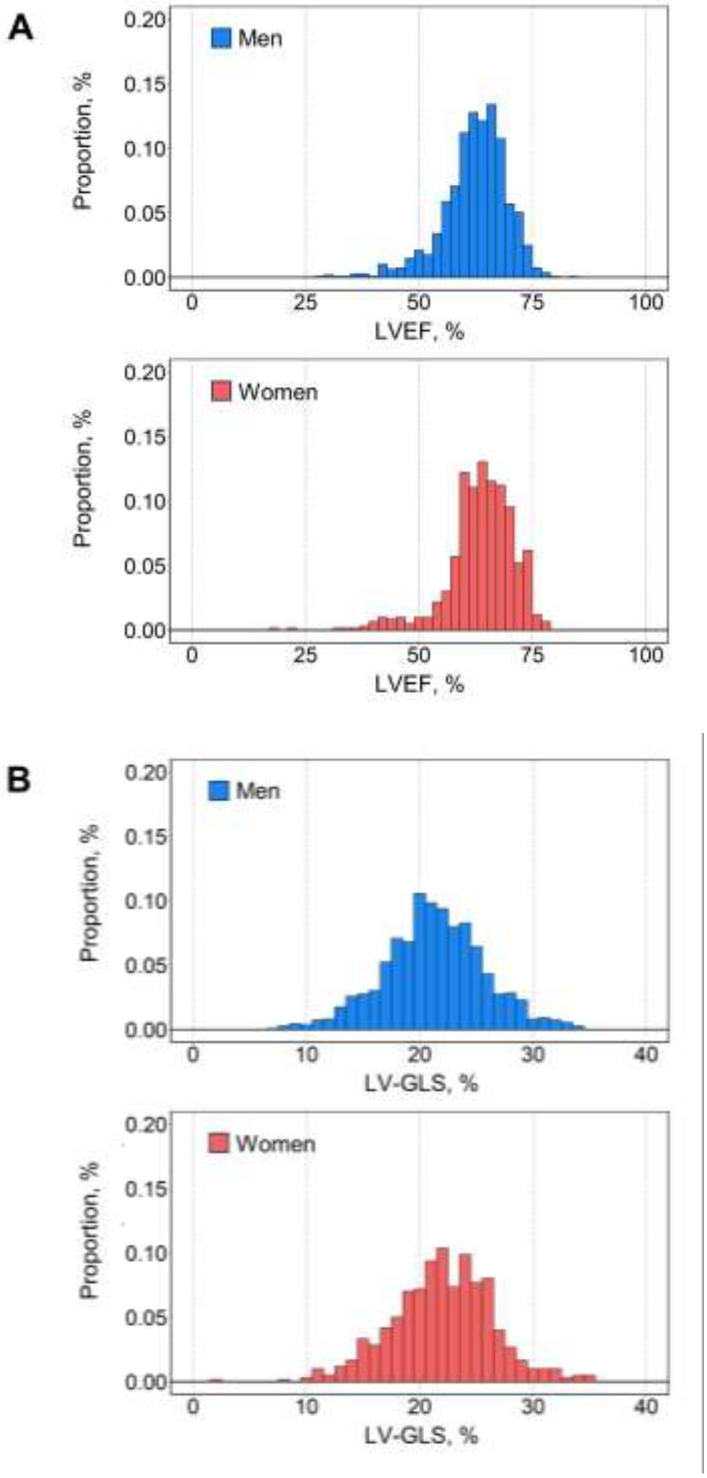


LVEF, left ventricular ejection fraction; LV-GLS, left ventricular global longitudinal strain

© 2025 Kwak S et al. *JAMA Network Open*.

**eFigure 3. Mortality according to sex in the entire cohort and the propensity-matched cohort**

Survival free from all-cause mortality in (**A**) the entire population and (**B**) the propensity-matched cohort.

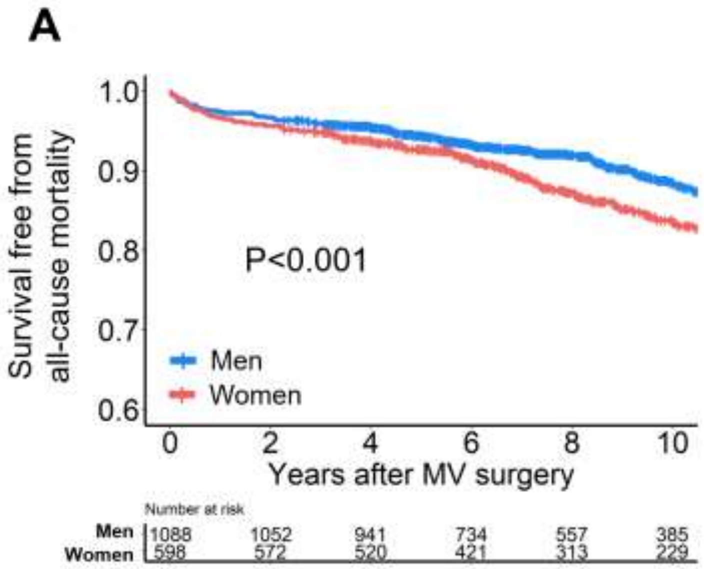


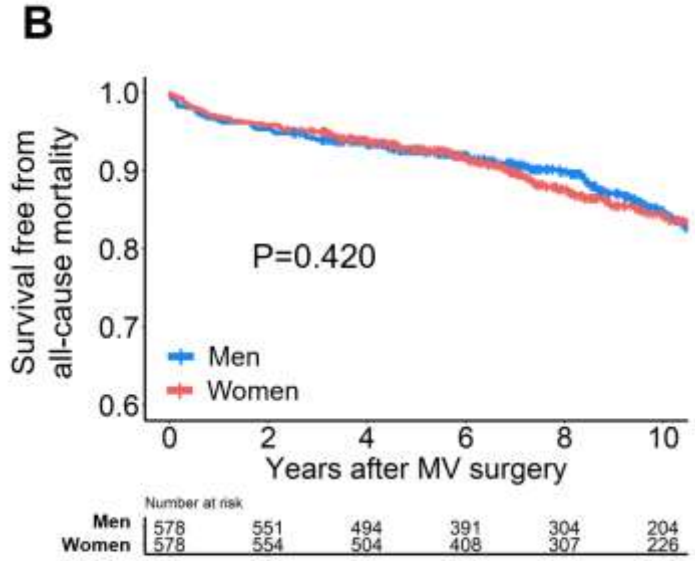


MV, mitral valve

© 2025 Kwak S et al. *JAMA Network Open*.

**eFigure 4. Mortality according to left ventricular systolic function and sex stratified by the different time period**

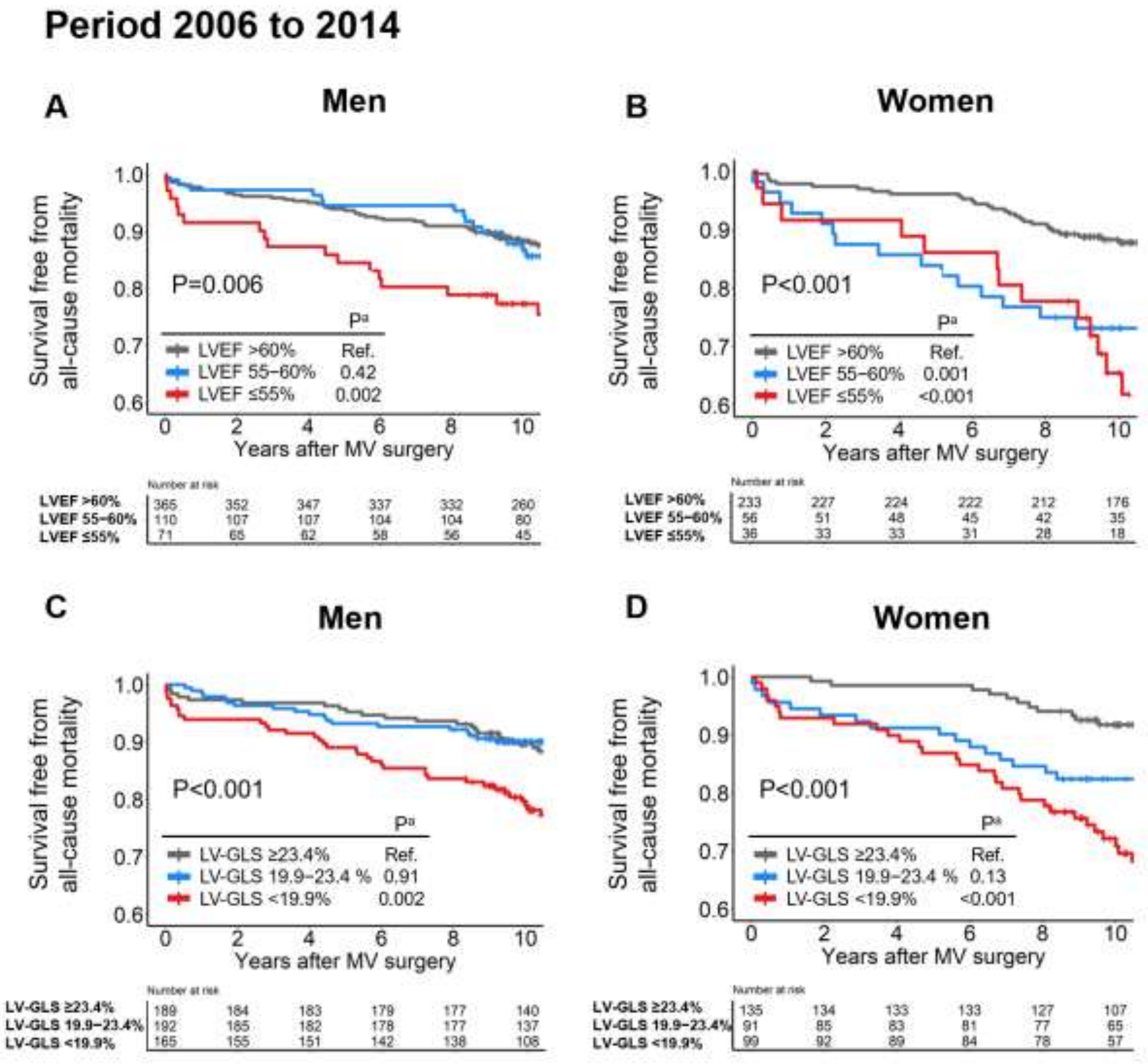


© 2025 Kwak S et al. *JAMA Network Open*.

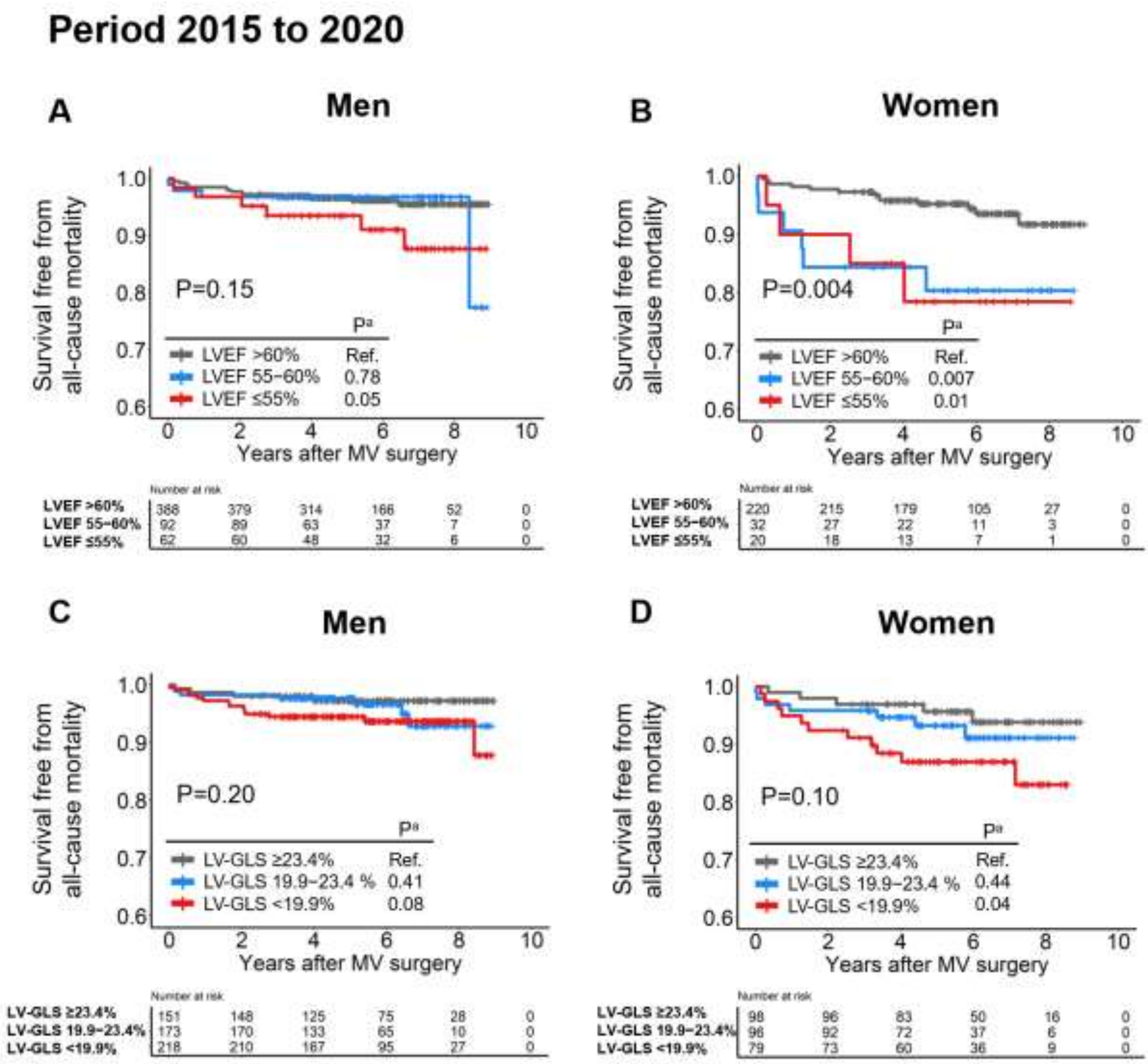


[a]P value for pairwise comparison with the LVEF >60% or LV-GLS ≥23.4%

LVEF, left ventricular ejection fraction; LV-GLS, left ventricular global longitudinal strain

© 2025 Kwak S et al. *JAMA Network Open*.

**eFigure 5. Mortality according to left ventricular systolic function and sex in the propensity-matched cohort**

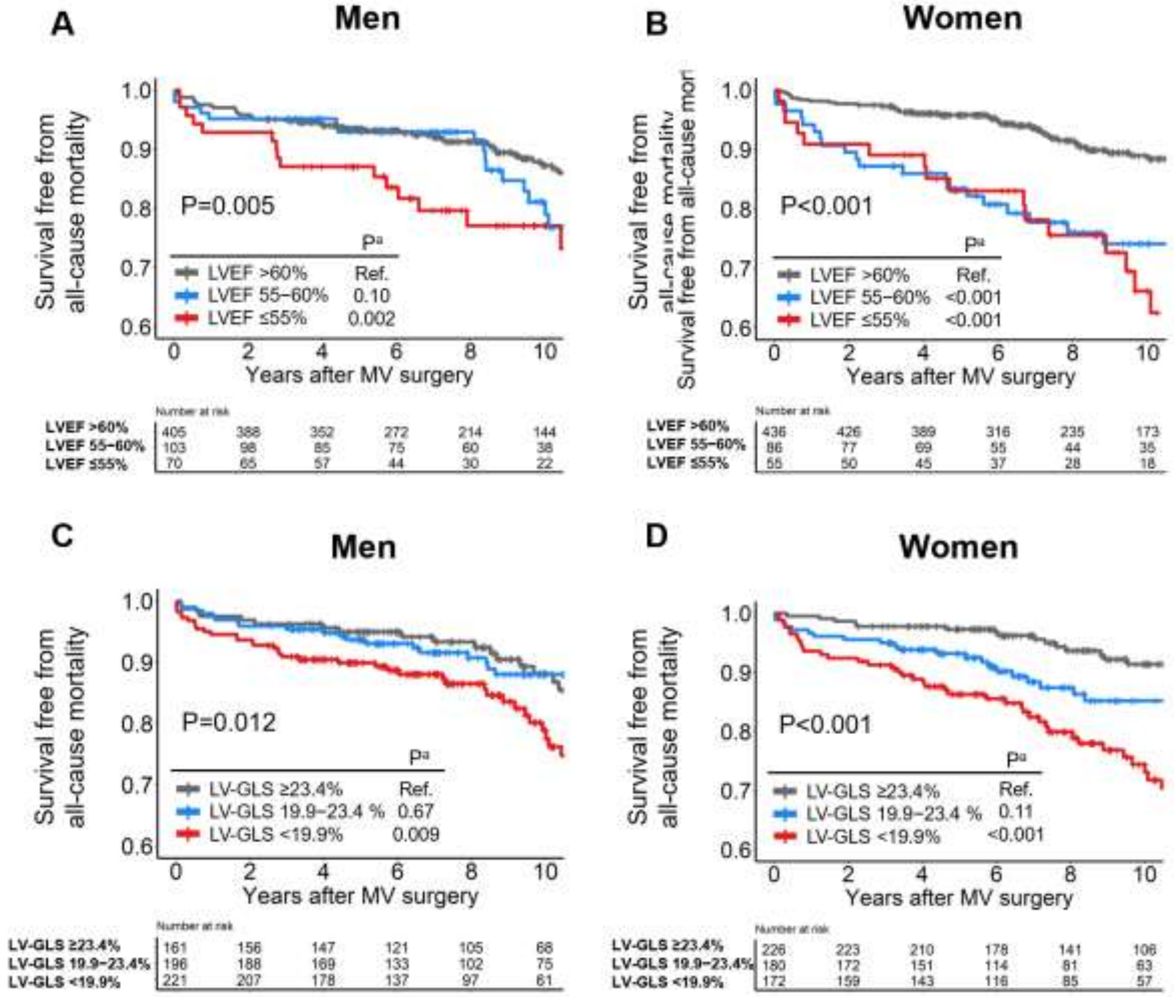


[a]P value for pairwise comparison with the LVEF >60% or LV-GLS ≥23.4%

LVEF, left ventricular ejection fraction; LV-GLS, left ventricular global longitudinal strain

© 2025 Kwak S et al. *JAMA Network Open*.

**eFigure 6. Mortality according to left ventricular systolic function and sex in asymptomatic individuals**

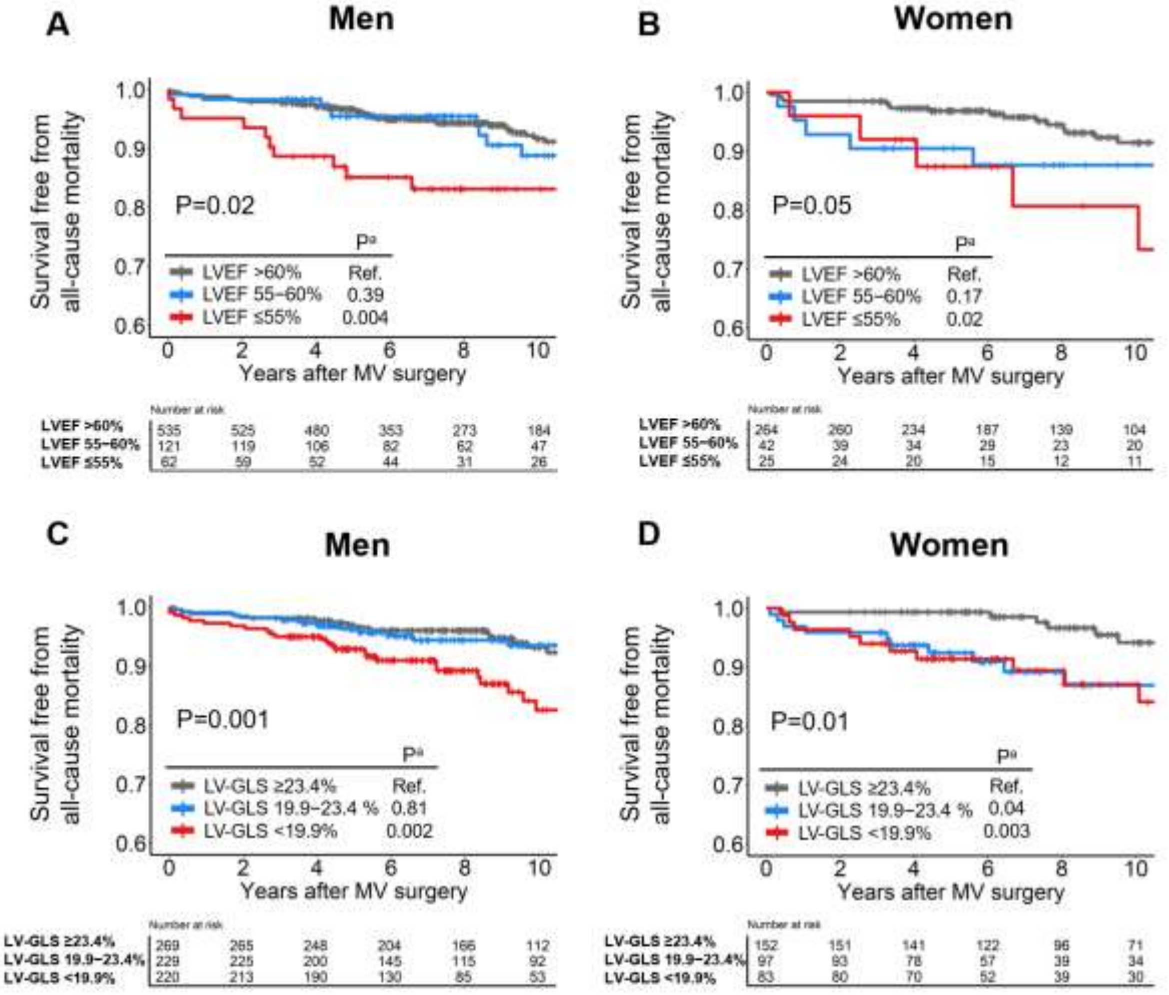


LVEF, left ventricular ejection fraction; LV-GLS, left ventricular global longitudinal strain; MV, mitral valve

**eFigure 7. Relationship between left ventricular systolic function and mortality by sex in asymptomatic individuals without evidence of obstructive coronary artery disease**
Obstructive CAD was defined as the absence of a history of myocardial infarction and no concomitant CABG.

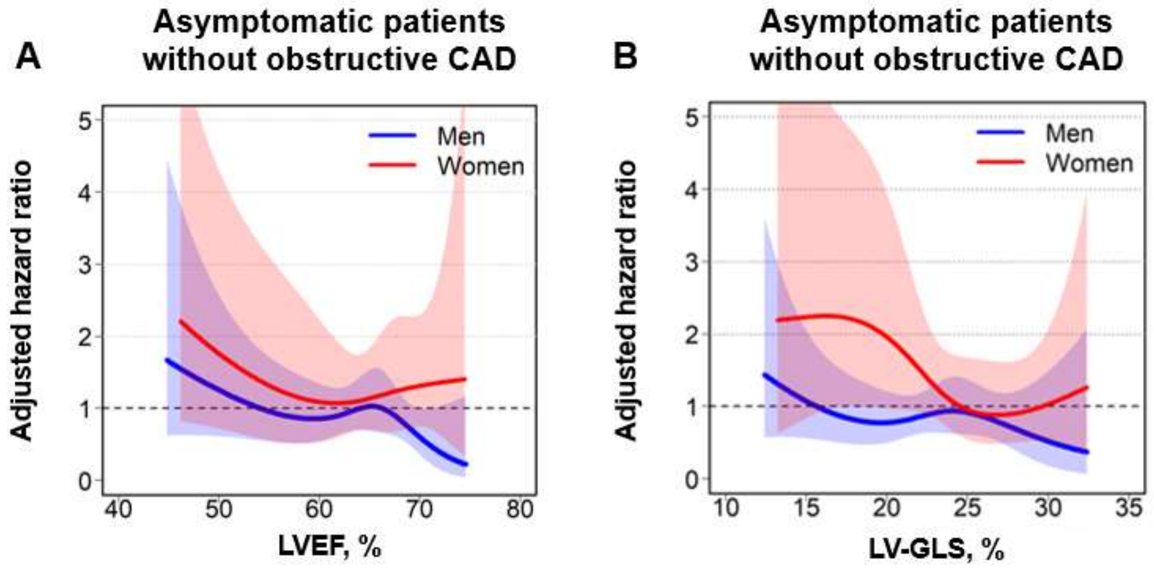


CABG, coronary artery bypass grafting; CAD, coronary artery disease, LVEF, left ventricular ejection fraction; LV-GLS, left ventricular global longitudinal strain

© 2025 Kwak S et al. *JAMA Network Open*.
