# Supplementary material for: Sex-Specific Association of Left Ventricular Function With Mortality in Severe Mitral Regurgitation
Source: JAMA Netw Open. 2025 Mar 31;8(3):e252420. doi: 10.1001/jamanetworkopen.2025.2420 (PMC11959442; doi:10.1001/jamanetworkopen.2025.2420)
Supplement: Supplement 2. — Data Sharing Statement [file jamanetwopen-e252420-s002.pdf]

Data Sharing Statement

Kwak. Sex-Specific Association of Left Ventricular Function With Mortality in Severe Mitral Regurgitation. *JAMA Netw Open*. Published March 31, 2025.
doi:10.1001/jamanetworkopen.2025.2420

Data
Data available: Yes
Data types: Deidentified participant data
How to access data: The data supporting the findings of this study will be made available upon reasonable request, subject to institutional and ethical guidelines, by contacting the corresponding authors directly.
When available: With publication

Supporting Documents
Document types: None

Additional Information
Who can access the data: Researchers requesting the data with a reasonable research objective
Types of analyses: Statistical analysis
Mechanisms of data availability: The data will be shared after approval by the institutional review board of the study centers.
